# Screening and Counseling for Unhealthy Alcohol Use in Primary Care Practices

**DOI:** 10.1001/jamanetworkopen.2025.53518

**Published:** 2026-01-22

**Authors:** Daniel E. Jonas, Sean R. Riley, Leslie Brouwer, Marcella H. Boynton, Colleen Barclay, Debbie Grammer, Chris Weathington, Mary McCaskill, Sarah A. Birken, Kimberly Shoenbill, Adam J. Zolotor, Samuel Cykert, Darren A. DeWalt

**Affiliations:** 1 Division of General Internal Medicine, Department of Internal Medicine, Ohio State University College of Medicine, Columbus; 2 Department of Medicine, School of Medicine, University of North Carolina Chapel Hill, Chapel Hill; 3 North Carolina Area Health Education Center, Chapel Hill; 4 Department of Implementation Science, Wake Forest University School of Medicine, Wake Forest, North Carolina; 5 Department of Internal Medicine and Clinical Informatics Program, Moffitt Cancer Center, Tampa, Florida; 6 Department of Family Medicine, School of Medicine, University of North Carolina, Chapel Hill

## Abstract

**Importance:**

Unhealthy alcohol use is a leading cause of preventable deaths and is associated with many societal and health problems. Fewer than one-third of people who visit primary care practices in the US are asked about or ever discuss alcohol use with a health professional.

**Objective:**

To evaluate the association between primary care practice facilitation and adoption of evidence-based screening and brief counseling for unhealthy alcohol use.

**Design, Setting, and Participants:**

The Stop Unhealthy Alcohol Use Now (STUN) single arm, multi-site implementation study was performed at 21 primary care practices across North Carolina between February 1, 2020, and September 1, 2023.

**Intervention:**

Enrolled practices received 12 months of the practice facilitation implementation strategy, including quality improvement coaching, electronic health record support (eg, creating smart phrases or flowsheets, retrieving data), and clinician training on screening and counseling for unhealthy alcohol use.

**Main Outcomes and Measures:**

Implementation outcomes reflected adoption of evidence-based screening and counseling, including number and percentage of adult patients who were screened for unhealthy alcohol use and who received brief counseling after a positive screening result.

**Results:**

The 21 practices served 54 294 adult patients (mean [SD], 3386.2 [3418.0] per practice). Mean screening rates increased significantly, from 17.4% (95% CI, 6.0%-28.9%) per practice to 57.6% (95% CI, 29.1%-86.1%) by the end of the second quarter of practice facilitation (primary outcome; *P* < .001). Among screened patients, a weighted 13.9% (95% CI, 6.8%-21.1%) had positive results. The percentage of adult patients with documentation of receiving brief counseling after a positive screening result increased from 0 to 32.3% (95% CI, 13.3%-51.4%) by the end of the second quarter of practice facilitation (*P* < .001). After month 6, assessment of the implementation outcomes showed sustainment. There was significant variability across participating practices for screening and counseling outcomes.

**Conclusions and Relevance:**

The findings of this quality improvement study suggest that practice facilitation was associated with increased adoption of evidence-based screening and counseling for unhealthy alcohol use when provided to small and medium-sized primary care practices. This increase is projected to substantially reduce the harms of unhealthy alcohol use.

## Key Points

**Question:**

Does practice facilitation provided to small and medium-sized primary care practices increase the number and percentage of patients who are screened and counseled for unhealthy alcohol use?

**Findings:**

In this quality improvement study of 21 practices and 54 294 patients, rates of screening and brief counseling increased significantly, and improvements were sustained, despite COVID-19 pandemic–related barriers. There was significant variability in adoption of evidence-based screening and counseling across participating practices.

**Meaning:**

These findings suggest that practice facilitation was associated with increased adoption of evidence-based screening and counseling for unhealthy alcohol use.

## Teaser

This quality improvement study evaluates the association between primary care practice facilitation and evidence-based screening, counseling, and pharmacotherapy for unhealthy alcohol use among primary care patients.

## Introduction

Unhealthy alcohol use is very common, affecting approximately one-third of adults in the US, is among the top 3 causes of preventable deaths in the US (accounting for approximately 178 000 deaths/y), and is associated with many societal and health problems, including cancers (eg, oral, esophageal, colorectal, liver, breast), gastrointestinal problems (eg, cirrhosis, ulcers, pancreatitis, gastritis), cardiovascular problems (eg, hypertension, stroke), mental health problems (eg, depression, suicide, anxiety, cognitive impairment), birth complications, injuries, violence, and mortality.^1,2,3,4^ It is a key contributor to recent declines in US life expectancy, especially among middle-aged persons and those living in rural areas.^5,6^ Multiple systematic reviews and national recommendations have established that screening with brief validated questionnaires can accurately detect unhealthy alcohol use and that subsequent counseling in primary care delivered by a variety of clinician types can significantly reduce alcohol consumption.^7,8,9^ Effective counseling interventions often include motivational interviewing techniques, an effective patient-centered approach for achieving behavior change.^10,11^

Primary care visits provide a valuable opportunity to address unhealthy alcohol use because more than half of US adults have regular visits with primary care, and although trust in the health care system has decreased in recent years, most individuals continue to trust their own primary care clinicians.^12,13,14^ However, relatively few patients in the US ever discuss alcohol use with their primary care clinician.^15,16^ Some of the key barriers include lack of a formal process for screening and follow-through, lack of awareness of evidence-based screening and counseling techniques, competing priorities, limited skills in the delivery of counseling, limited access to pertinent services for persons identified to have alcohol use disorder (AUD), and awareness of limited evidence on referral to treatment strategies (in the context of primary care screening and brief intervention programs).^17,18,19,20,21^

Practice facilitation is an implementation strategy that has the potential to address and overcome key barriers to implementation (eg, lack of a formal process, limited counseling skills). Our method of practice facilitation follows the approach used in the EvidenceNow initiatives supported by the Agency for Healthcare Research and Quality (AHRQ).^22^ We selected this approach because members of our team have successfully used this approach in prior work, our workforce was already skilled in the relevant processes, and it is an evidence-based implementation strategy.^23,24,25^ We used a quality improvement approach to guide change, helping practices with workflow analysis and redesign, electronic health record (EHR) support (eg, using available tools, creating smart phrases or flowsheets, retrieving data), and use of the Model for Improvement.^26,27^ A growing body of evidence indicates that practice facilitation is an effective strategy for implementing evidence-based practices. For example, 2 systematic reviews including 41 total unique studies^28,29^ reported an increased likelihood of adopting evidence-based guidelines among primary care practices that received practice facilitation relative to those who did not and an improvement in various chronic disease care measures for those who received practice facilitation. However, none of the studies in either review focused on unhealthy alcohol use.

We designed the Stop Unhealthy Alcohol Use Now (STUN) study to evaluate the association between primary care practice facilitation and evidence-based screening, counseling, and pharmacotherapy for unhealthy alcohol use. This report focuses on the first aim of the STUN study, which sought to evaluate the association between primary care practice facilitation and adoption of evidence-based screening and brief counseling for unhealthy alcohol use.

## Methods

### Study Design

The STUN study was registered in Clinicaltrials.gov^30^ and the STUN design has been described in a previous publication.^31^ Herein, we provide the results for the main hypotheses for aim 1 of the study. The present quality improvement study was an implementation study in which all enrolled practices received the practice facilitation implementation strategy for a 12-month period. This study was reviewed and approved by the University of North Carolina Institutional Review Board, and all participants provided online consent prior to taking the survey. We followed the Standards for Quality Improvement Reporting Excellence (SQUIRE) reporting guideline. Our primary hypotheses were that practice facilitation would be associated with an increase in the number and percentage of patients in a practice who were (1) screened for unhealthy alcohol use, (2) identified to have unhealthy alcohol use, and (3) provided with brief counseling.

### Setting and Participants

Primary care practices in North Carolina were eligible for study enrollment if 10 or fewer physicians and advanced practice professionals occupied a single clinic location and did not currently or previously receive facilitation services specifically related to unhealthy alcohol use. Practices were recruited by our practice facilitators (ie, practice coaches) on a virtual basis using phone calls, emails, and video conferencing. To assist with recruitment, the research team provided informational materials (eg, covering the study description, importance of addressing unhealthy alcohol use, potential benefits of participation), scheduled informational webinars with practice representatives, and participated in regular educational sessions with practice facilitators. Decisions to participate were typically made through consensus discussions with practice leaders and clinical champions (eg, medical directors, practice managers, physicians, nurse practitioners). Enrolled practices agreed to: (1) work with practice facilitators to implement an evidence-based unhealthy alcohol use screening process as well as a process for counseling and/or referring patients with positive screening results; (2) participate in webinars conducted by project personnel about the screening and brief counseling process as well as how and when to prescribe medications for AUD; (3) respond to surveys about the practice environment and the improvement process; and (4) collect and report monthly implementation data with help from practice facilitators. Study involvement lasted a total of 21 months (consisting of a 3-month baseline data collection phase, 12-month implementation phase, and 6-month postimplementation sustainment phase). Practices were enrolled and started the 12-month implementation phase on a rolling basis, with the first practice starting February 1, 2020, and the last practice starting March 1, 2022; the study was completed September 1, 2023. It should be noted that the US Secretary of Health and Human Services declared COVID-19 a public health emergency in January 2020, meaning that clinic recruitment and training, as well as collection of adoption measures, overlapped with the peak and immediate aftermath of the COVID-19 global pandemic.^32^

### Practice Facilitation for Implementation of Screening and Counseling

Enrolled practices received 12 months of practice facilitation (the implementation strategy), including quality improvement coaching, EHR support (eg, using available tools, creating smart phrases or flowsheets, retrieving data), and training on screening and counseling for unhealthy alcohol use. Evidence-based interventions included implementation of screening and counseling. Further details are provided in the published protocol.^31^ In brief, facilitation followed the approach used in the EvidenceNow initiatives supported by the AHRQ and used the Model for Improvement, an integrated approach to process improvement that has been shown to deliver quick and substantial results in both quality and productivity in diverse settings.^22,26,27^ Practices received as many as 2 hours of facilitation services per month. They were expected to use a Plan-Do-Study-Act approach, applying tests of change fairly independently, but with coaching from a member of the facilitation team.^33^ Facilitators aimed to ensure that practices had established specific evidence-based workflows to screen for unhealthy alcohol use, such as those described in the video training modules available on our website and on YouTube.^34,35,36,37,38^ Participating practices were required to use a validated screening tool (the Single Alcohol Screening Question [SASQ], Alcohol Use Disorders Identification Test [AUDIT], or AUDIT-Consumption [AUDIT-C]).^39,40,41^ Our team recommended the use of a brief initial screen with 3 or fewer questions (either the SASQ or the AUDIT-C, depending on availability in their EHR) followed by the use of the full AUDIT (10 questions) for screening-related assessment for those with a positive initial screen (to help ascertain whether an individual likely had an AUD). We recommended brief counseling for all patients with positive screening results based on standard cutoffs on the validated brief initial screening questions, specifically, (1) a “yes” response to having 5 or more (for men younger than 65 years) or 4 or more (for all women and men 65 years and older) drinks in a day if they were administered the SASQ or (2) a score of 4 or more (for men) or 3 or more (for women) on the AUDIT-C. Facilitators conducted periodic data checks to ascertain progress, sharing the results of these data checks with the practice using audit and feedback methods. Webinars and video recordings served as additional tools that facilitators used to educate participants.^34,35,36,37,38,42^ Expert consultation with physician faculty was made available, primarily in a virtual format, to supplement facilitation efforts. Although practice facilitation was initially planned to primarily involve on-site face-to-face meetings with modest use of email, telephone, and video communication, remote communication was ultimately the primary means of communication due to the effects of the COVID-19 pandemic and the risks of exposure associated with on-site face-to-face contact.

The STUN facilitation team included leaders and staff of the North Carolina Area Health Education Centers (AHEC) Practice Support Program, a program with permanent statewide infrastructure that includes personnel trained to support and deliver practice facilitation services. The North Carolina AHEC has developed strong relationships with primary care practices across the state.^43^ The existing relationships can expedite recruitment and increase the efficiency of communications. Facilitators were trained in 49 coaching competencies, and training content was continually updated to help ensure that facilitators were prepared to help practices respond to emergent needs as they arose. Additional details describing the approach to facilitation meetings were reported in the published protocol.^31^

### Outcomes and Measures

The primary outcomes were implementation measures reflecting adoption (ie, uptake, use) of evidence-based screening and counseling, consisting of the change in number (ie, absolute count) and percentage of adult patients (1) who were screened for unhealthy alcohol use and (2) who received brief counseling after a positive screening result. Data regarding the number and percentage of patients in the target population who were screened for unhealthy alcohol use, had positive screening results, and received brief counseling were collected via data forms on the study website, medical record review, or directly from the practices’ EHR. To ensure patient confidentiality and in keeping with a quality improvement approach, alcohol screening data (eg, number of patients screened) were aggregated at the practice level and then submitted to the research team. The research team did not have access to individual patient-level data.

### Data Collection

The study collected multiple types of data related to the potential association between practice facilitation and implementation of evidence-based alcohol screening and counseling. These data types are detailed in the following sections.

#### EHR

Practices obtained data for implementation outcomes using their EHR system or by recording the data in a registry, the creation of which was guided by the practice facilitators and project team (including D.E.J., C.B., D.G., C.W., and M.M.). For example, when available, monthly data on delivery of counseling was ascertained based on documentation in the EHR in structured data fields or using searchable smart phrases entered in progress notes. These data were entered into the analytic dataset as aggregate patient counts, and from these data, percentages screened for unhealthy alcohol use and receiving brief counseling after a positive screening result were calculated for each practice. Practices were instructed to submit data for screening and counseling numbers and rates each month using unique patients in the denominator. This approach ensured that no protected health information was included in the dedicated online tool used to generate the project database. Monthly implementation measures were collected for the 3-month period prior to the implementation phase (ie, baseline), during the implementation phase for 12 months, and the 6-month postimplementation follow-up (ie, 18 months after baseline). This approach yielded 7 consecutive 3-month (quarterly) increments that covered a total time span of 21 months.

#### Medical Record Review

Practice facilitators were unable to obtain monthly implementation data from the EHRs or registries for 5 of the study practices. These 5 practices were not significantly different from other included practices by demographics or patient characteristics, although they differed by not having either the EHR capabilities for automated data extraction or the staff with the expertise to conduct such data extraction. In these cases, small (1- to 3-member) teams of experienced practice facilitators visited each practice and reviewed patient medical records from each month of the implementation phase and the 6-month postimplementation period to garner the necessary practice-level outcomes data, which were then entered into the analytic dataset as aggregate patient counts.

#### Clinician and Staff Surveys

Data regarding contextual factors, including practice characteristics, patient population, and EHR capabilities, were obtained via staff and clinician surveys administered at baseline, at the end of implementation (12 months after baseline), and 6 months after implementation (18 months after baseline). As many as 5 clinicians and staff members were invited to complete surveys, depending on the size of the practice. Practice characteristics, including patient demographic composition, were obtained from baseline surveys completed by practice staff, who provided aggregated estimates from their EHR databases when available (eg, race, ethnicity, payer mix, and age distribution). Race and ethnicity were self-reported by patients and included American Indian or Alaska Native, Asian, Black or African American, Native Hawaiian or Other Pacific Islander, White, multiracial or other, or unknown race and Hispanic or Latino ethnicity; these data were collected to provide descriptive information about the patients served by the participating practices.

#### Facilitator Logs

Practice facilitators were instructed to track each of their interactions with each practice. These logs included the mode of delivery (video, in-person, email, or phone) and the duration of contact for each facilitation interaction.

### Statistical Analysis

For describing the patient populations seen within the participating clinics, we provide unweighted and weighted data when applicable (for presenting age, sex, race, ethnicity, and payer). Weighting was based on the volume of patients served by each clinic. Using Stata, version 15 (StataCorp LLC), we used aweight with the summarize command to compute variance as the weighted sum of squared deviations divided by n − 1, giving greater influence to higher-precision observations while retaining standard degrees of freedom. With the tabulate command, aweight generates weighted counts and percentages, treating each observation as repeated according to its weight.

For each participating clinic, monthly data representing the number of adult patients seen in the clinic, screened for unhealthy alcohol use, identified to have unhealthy alcohol use, and provided with brief counseling were collected. As previously noted, these clinic-level data were collected for each month during the baseline period (ie, 3 months prior to initiation of the implementation phase), during the practice facilitation implementation phase (ie, for 12 months), and to assess postimplementation sustainment (ie, 6 months post implementation). Using the count data for numbers of adult patients seen in the practices, screened for unhealthy alcohol use, identified to have unhealthy alcohol use, and provided with counseling, percentages were calculated monthly. We then computed a repeated-measures analysis of variance (ANOVA) for the change (1) from baseline to the second quarter (the a priori primary outcome of the study) and (2) an overall test of change. Time was modeled using longitudinal trajectory modeling. Specifically, using the repeated monthly count data, we computed population mean models (in the case of screening percentage models, we used a Poisson distribution; for the other outcomes, we used a negative binomial) with repeated monthly measures (level 1) nested within clinics (level 2) to test whether mean counts for screening, identification, and counseling increased (1) from baseline to the end of the second quarter (the prespecified time point for the primary outcome) and (2) across the 7 quarters of study data. This generalized estimating equation (GEE) modeling approach accounts for the nonindependent data structure of the repeated measures (monthly) data by appropriately adjusting the SEs.^44^ Clinic size was accounted for by incorporating the count of monthly patient encounters as an offset variable, thereby adjusting model estimates for the number of patients available to be screened or referred monthly at each clinic. For each outcome, we iteratively tested the random intercept, linear, quadratic, and cubic time models. The time variable itself was coded by month, with values ranging from 0 to 20. Statistical significance of effects, coupled with comparative model fit testing (log-likelihood ratio, χ^2^ difference testing) was used to select the best fitting models. The overall test of change is reflected in the statistically significant *P* values generated by these models,^44^ with 2-sided *P* < .05 indicating statistical significance.

## Results

**Figure 1.**
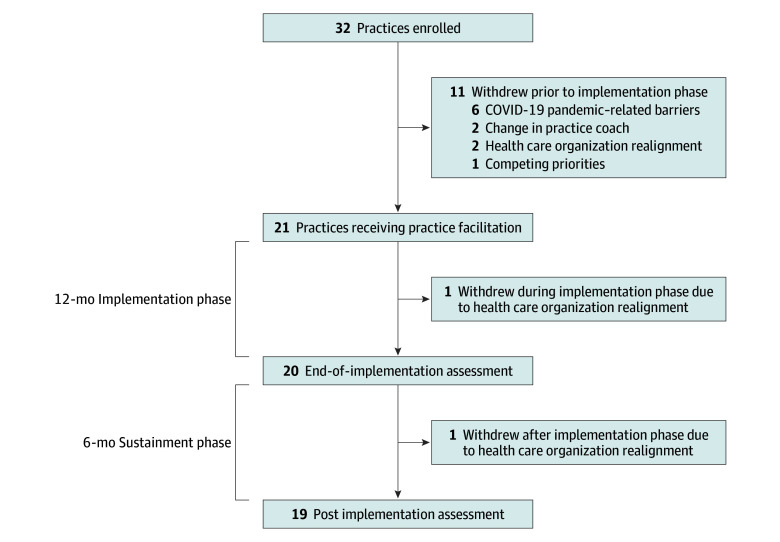
Practice Enrollment, Retention, and Timing of Assessments in the Stop Unhealthy Alcohol Use Now Study

A total of 32 practices were initially enrolled, and 21 remained in the study to receive practice facilitation and reported baseline data and data for our implementation measures (such as data for monthly screening rates). Eleven practices (34.4%) withdrew prior to the implementation phase; reasons for practice withdrawals are provided in Figure 1. Most of those withdrawals were due to COVID-19 pandemic–related barriers. Twenty practices completed the 12-month assessment and 19 completed the postimplementation (sustainment) assessment. During the implementation phase, practice facilitators had a total of 280 facilitation encounters (mean, 15.5 [range, 5-28] encounters per practice) with practice staff and clinicians. Of these, 139 (49.6%) were delivered by video conferencing, 72 (25.7%) by email, 62 (22.1%) in person, and 7 (2.5%) by phone. Video encounters lasted a mean of 34.0 (range, 15.0-70.0) minutes, while in-person encounters lasted a mean of 60.3 (range, 15.0-240.0) minutes.

### Participating Practices

The mean (SD) number of adult patients per practice was 3386.2 [3418.0] and the mean (SD) number of weekly adult patient visits was 315.0 (432.8); most practices had 2 to 5 clinicians, most were single-specialty practices, and most were clinician-owned practices (Table 1). Ten practices (47.6%) had at least 1 behavioral health clinician working in the practice. For payer mix, a weighted mean (SD) 34.2% (14.7%) of patients were covered by Medicare, 16.2% (10.6%) were covered by Medicaid, 4.0% (5.2%) had dual eligibility, 38.0% (13.8%) were covered by private or commercial insurance, and 7.6% (3.3%) were not insured. A total of 54 294 patients were served by the participating practices. Weighted data (by number of patients in the practice) indicate that most patients were aged either 40 to 59 years or 60 to 75 years; a mean (SD) 51.7% (5.1%) were female and 37.7% (8.7%) were male. In terms of race, a mean (SD) of 0.1% (0.3%) of patients identified as American Indian or Alaska Native; 0.4% (0.5%), Asian; 40.1% (14.0%), Black or African American; 0.1% (0.2%), Native Hawaiian or Other Pacific Islander; 52.0% (14.4%), White; 3.0% (3.5%), multiracial or other race; and 6.0% (3.6%), unknown race. Weighted data indicated that a mean (SD) of 7.0% of patients (8.8%) identified as being of Hispanic or Latino ethnicity.

**Table 1.** Baseline Characteristics of Practices and Patients

Characteristic	Unweighted data	Weighted data by patient number in practice
**Practices (N = 21)**		
No. of patients per practice, mean (SD)	3386.2 (3418.0)	NA
No. of weekly patient visits, mean (SD)	315.0 (432.8)	NA
Practice size, No. (%)		
Solo practice	3 (14.3)	NA
2-5 Clinicians	14 (66.7)	
6-10 Clinicians	3 (14.3)	
11-15 Clinicians	1 (4.8)	
Practice specialty mix, No. (%)		
Single specialty	18 (85.7)	NA
Multispecialty	3 (14.3)	
Practice type, No. (%)		
Clinician-owned	11 (52.4)	NA
Hospital-owned	2 (9.5)	
FQHC or look-alike	5 (23.8)	
EHR company, No. (%)		
Epic Systems Inc	6 (28.6)	NA
athenahealth	4 (19.0)	
eClinicalWorks	4 (19.0)	
Allscripts	3 (14.3)	
AdvancedMD	1 (4.8)	
Kareo	1 (4.8)	
Meditab	1 (4.8)	
Other or not reported	1 (4.8)	
Has someone who can configure or write quality reports from the EHR or EMR, No. (%)	8 (38.1)	NA
Has a behavioral health clinician, No. (%)	10 (47.6)	NA
**Patients (N = 54 294)**		
Age, mean (SD), %		
0-17 y	6.5 (7.2)	11.4 (7.8)
18-39 y	19.1 (6.4)	21.5 (5.0)
40-59 y	30.8 (9.7)	27.5 (6.7)
60-75 y	30.0 (10.5)	26.9 (8.5)
≥76 y	13.7 (8.2)	13.0 (5.4)
Sex, mean (SD), %		
Male	39.0 (7.4)	37.7 (8.7)
Female	54.3 (4.8)	51.7 (5.1)
Race, mean (SD), %		
American Indian or Alaska Native	0.4 (0.7)	0.1 (0.3)
Asian	0.5 (0.7)	0.4 (0.5)
Black or African American	37.8 (15.3)	40.1 (14.0)
Native Hawaiian or Other Pacific Islander	0.3 (0.6)	0.05 (0.2)
White	54.4 (16.2)	52.0 (14.4)
Multiracial or other^a^	2.4 (3.3)	3.0 (3.5)
Unknown	6.6 (4.9)	6.0 (3.6)
Latino or Hispanic ethnicity, mean (SD), %	7.5 (8.0)	7.0 (8.8)
Payer, mean (SD), %		
Private insurance	36.9 (14.7)	38.0 (13.8)
Medicare only	33.5 (14.9)	34.2 (14.7)
Medicaid only	15.7 (12.7)	16.2 (10.6)
Dual insurance	6.4 (6.8)	4.0 (5.2)
No insurance	7.4 (4.4)	7.6 (3.3)

Abbreviations: EHR, electronic health record; EMR, electronic medical record; FQHC, federally qualified health center; NA, not applicable.

^a^ Includes patients categorized as multiracial or other race as recorded in practice data; no further subcategories were specified.

### Number and Percentage of Patients Screened

**Figure 2.**
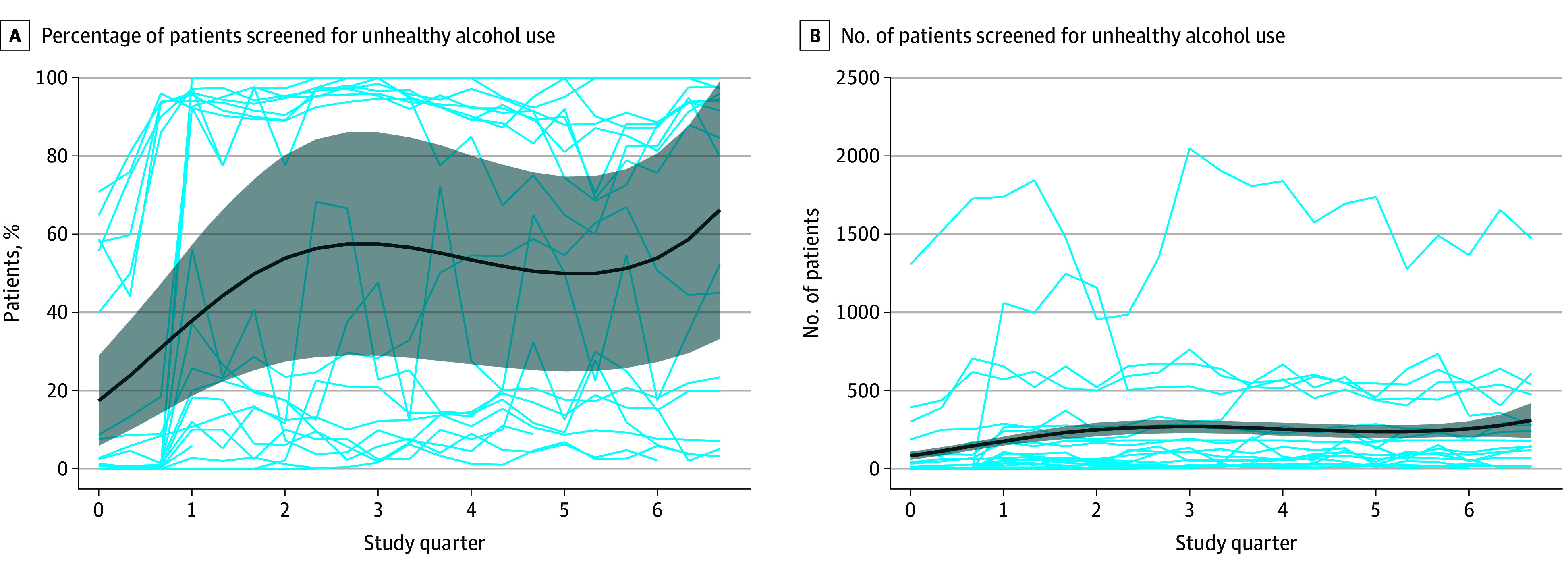
Number and Percentage of Patients Screened for Unhealthy Alcohol Use The percentage represents patients who had documentation of screening among those adults who had an encounter and were due for screening. The thin light blue lines represent patient counts or percentages for each clinic. The thick dark blue lines represent the estimated model; gray shading represents 95% CIs.

The repeated-measures ANOVA within-participants effect using the Huynh-Feldt correction for sphericity showed a significant change over time in the number of patients screened during the 3 months prior to and 6 months after the implementation phase began (F_1,18_ = 3.96; *P* = .042). The weighted results for percentage of patients screened for unhealthy alcohol use along with unweighted counts for the number screened are shown in Table 2. Screening using validated tools for the identification of unhealthy alcohol use (AUDIT-C, SASQ, or AUDIT) increased significantly from baseline to the second quarter (our primary outcome), from a mean (SD) of 435 (1037) to 690 (892) adults per quarter per practice. The model-estimated mean screening rates were 17.4% (95% CI, 6.0%-28.9%) at 3 months prior to the implementation and increased to 57.6% (95% CI, 29.1%-86.1%) at 6 months after baseline (*P* < .001) (Figure 2). The increase in screening generally occurred quickly, within the baseline period (as practices were preparing to ramp up), or shortly after baseline (Figure 2). The increase in screening counts and percentages (compared with baseline) was sustained for the duration of the postimplementation follow-up (Table 2 and Figure 2). Of those patients who were screened, the number with positive screening results increased from baseline to the second quarter from a mean of 0 to nearly 62 adults per quarter per practice, and modeling estimated that a mean of 13.9% (95% CI, 6.8%-21.1%) had a positive screen result (eFigure 1 in Supplement 1). The percentage of patients with positive screen results was highest in the second and third quarters and remained below 20% throughout the data collection period (eFigure 1 in Supplement 1).

**Table 2.** Patient Screening and Counseling for Unhealthy Alcohol Use

Study quarter	Screening		Counseling	
	Patients screened, mean (95% CI), %^a^	No. of patients screened, mean (SD)	Patients counseled, mean (95% CI), weighted %^a^	No. of patients counseled, mean (SD)
0	34.2 (16.8-51.6)	435.4 (1037.3)	0 (NA)	0 (NA)
1	62.2 (43.7-80.7)	796.7 (1268.8)	33.0 (15.0-51.0)	16.1 (39.1)
2	63.6 (44.3-83.0)	690.0 (891.6)	32.3 (13.3-51.4)	16.3 (47.0)
3	65.1 (46.0-84.2)	864.4 (1331.2)	29.0 (8.9-49.0)	12.5 (35.5)
4	64.4 (47.5-81.3)	859.5 (1193.1)	26.6 (7.0-46.3)	13.1 (33.4)
5	62.9 (47.2-78.6)	831.4 (1084.0)	32.7 (11.9-53.5)	19.3 (44.3)
6	64.2 (46.5-81.8)	806.4 (1073.9)	58.6 (3.5-113.7)	38.6 (106.6)

Abbreviation: NA, not applicable.

^a^ Weighting was based on clinic patient volume.

### Number and Percentage of Patients Receiving Brief Counseling

**Figure 3.**
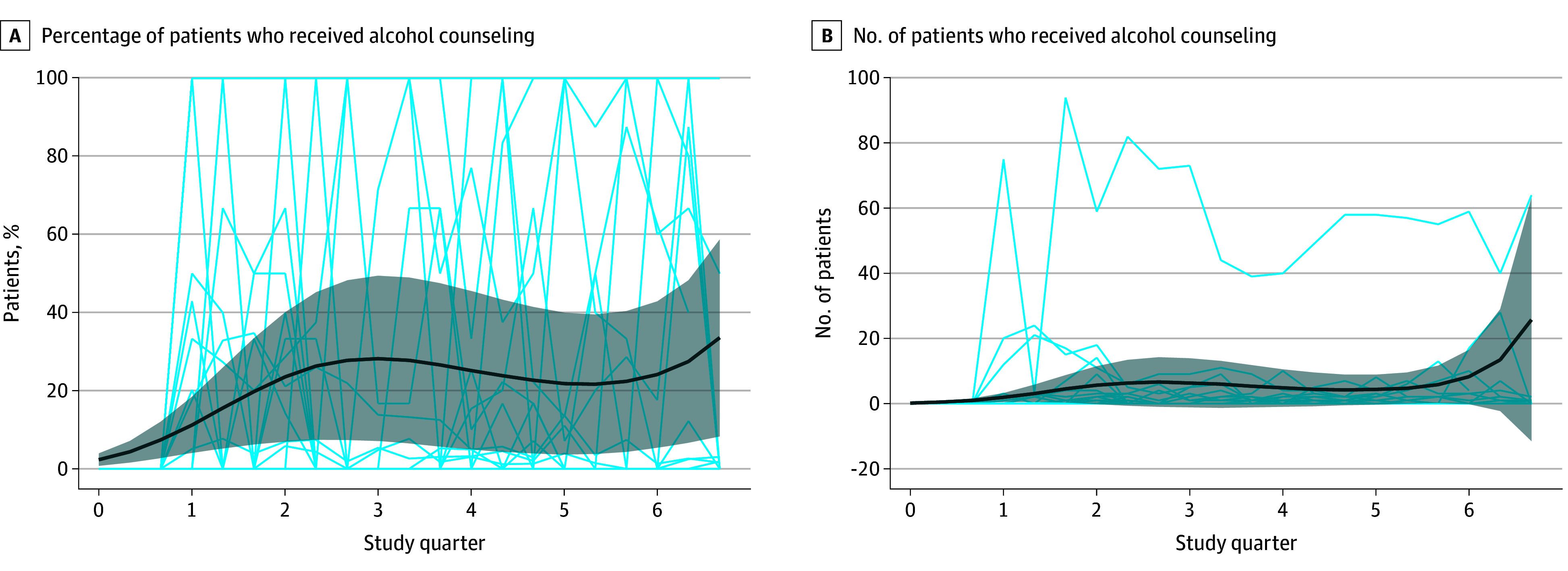
Number and Percentage of Patients Provided With Brief Counseling The percentage represents patients who had documentation of being provided with brief counseling when indicated (ie, of those with positive screening results). The thin light blue lines represent patient counts or percentages for each clinic. The thick dark blue lines represent the estimated model; gray shading represents 95% CIs.

At baseline, none of the practices were systematically providing or documenting evidence-based brief counseling interventions. The mean number of patients with documentation of receiving brief counseling after a positive screening result increased from 0 to 16.3 (SD, 47.0) adults per quarter per practice from baseline to the second quarter, and model estimated counseling rates increased from 0 per practice at baseline (before implementation) to a mean of 32.3% (95% CI, 13.3%-51.4%) at 6 months (*P* < .001) (Table 2 and Figure 3). After the second quarter, documentation of delivering brief counseling continued to increase for the third quarter; it then declined slightly although remained significantly above the baseline, showing sustainment (Table 2 and Figure 3).

### Heterogeneity of Changes in Screening and Counseling

There was significant variability across participating practices for screening and counseling outcomes. Looking at the monthly percentage screened for each practice, we observed a few patterns. Some practices started with a relatively high baseline rate and increased quickly to very high screening rates (>90%). A few practices started with very low baseline rates and increased to very high rates rapidly, some started very low and remained relatively low, and others exhibited gradual increases in their screening rates during the course of the study (Figure 2). For counseling, the monthly percentage of patients who received a brief intervention after a positive screening result varied widely over time within and across practices (Figure 3). Of note, the number of opportunities to provide counseling was fairly low in some practices, and a small change in the number of brief counseling interventions provided frequently resulted in a large change in the percentage. In sensitivity analyses, we stratified data by lower vs higher baseline screening rates (<50% vs ≥50% at baseline), demonstrating that those with higher baseline screening rates were generally able to achieve screening rates of 80% to 100% by the second quarter (eFigure 2 in Supplement 1). In contrast, those with lower baseline screening rates often remained at rates below 30%.

## Discussion

In this study, we observed significant improvements in the adoption (ie, uptake) of evidence-based screening and counseling for unhealthy alcohol use among primary care practices. Specifically, the model estimated screening rates more than tripled from baseline to the second quarter, from 17.4% of adult patients per practice to 57.6%. Similarly, the number of patients receiving brief counseling after a positive screening result rose from 0 to 32.3% of those with positive screening results, with a mean of 16 adults per quarter per practice. Exploratory analyses revealed variability among practices, with some reaching very high rates quickly, others showing gradual improvement, and some maintaining lower rates across the study period.

The STUN study achieved significant improvement in the main outcomes despite substantial barriers related to the COVID-19 pandemic (with the timing overlapping STUN’s recruitment and implementation phases), financial and staffing pressures on primary care practices during this time, and competing demands from a historic shift across North Carolina to Medicaid Managed Care, which placed large administrative demands on primary care practices during this time. We theorize that the increases in rates of screening and counseling found in STUN would have been larger without these barriers. In addition, our facilitators and primary care practices were faced with pivoting from on-site, in-person communications to relying primarily on video, remote communication methods (to avoid risks of exposure to and spreading of COVID-19).

Our study generated empirical evidence in support of the association between practice facilitation and improved implementation outcomes, adding to a growing body of literature on this topic. The STUN studywas 1 of 6 grantees funded by the AHRQ’s Evidence Now: Managing Unhealthy Alcohol Use Initiative to provide practice facilitation to primary care practices to implement screening and treatment recommendations from the US Preventive Services Task Force.^9,45^ To date, 2 other grantees have published their main findings. Both the Sustained Patient-Centered Alcohol-Related Care (SPARC) trial out of Washington State^46^ and the Unhealthy Alcohol Use initiative out of Virginia^47^ reported similarly positive findings on the uptake of screening and evidence-based treatments. Screening performance in STUN (mean, 57.6%) was lower than that observed in SPARC (83.2% for the practice facilitation group vs 20.8% for the control group; *P* < .001), but exceeded rates reported in the Virginia trial (35.5% for the practice facilitation group vs 1.4% for the control group; *P* < .001). Differences likely reflect contextual and design factors. For example, STUN enrolled smaller independent practices and had a longer facilitation period. Also, the timing of the COVID-19 pandemic relative to study phases was somewhat different. Our results are consistent with those of earlier studies, such as those by McNeely et al^48^ (which enrolled 6 primary care clinics) and Ornstein et al^49^ (which included 19 primary care practices), who demonstrated that workflow integration into EHRs and a practice-based quality improvement approach were feasible to implement, resulted in more frequent detection when screening was used at any primary care visit (vs screening at annual examinations only), and can lead to higher uptake of screening and brief intervention. In combination with our findings, these results reinforce the utility of practice facilitation in primary care settings and demonstrate its applicability beyond traditional chronic disease management to include delivery of evidence-based screening and interventions for behavioral health issues such as unhealthy alcohol use.

We specifically presented the results of individual practices together with the modeled means to illustrate the different responses to the intervention. Some of the most successful practices started with higher screening rates. In addition, some practices demonstrated substantial improvement in screening rates (but not in counseling rates) prior to the start of the formal practice facilitation implementation phase (ie, during baseline data collection). Before the formal practice facilitation started, our team had sessions with each practice to inform them of the measures, describe potential workflows and available EHR tools, and get agreement on proceeding. In other words, some elements of practice facilitation may have occurred while recruiting the practices. This is often necessary because participants frequently desire more detailed descriptions of workflows and toolkit elements before agreeing to participate. The Hawthorne effect might also account for some of the improvements observed during baseline data collection. Notably, some practices were able to increase screening efforts quickly with just that information. Future studies may consider deeper evaluation of the types of practices that benefit most from an ongoing practice facilitation strategy.

Historically, implementation efforts to increase the adoption of evidence-based screening and brief counseling for unhealthy alcohol use in primary care have relied on one-time training sessions, stand-alone educational programs, or EHR prompts.^50^ However, these approaches often failed to create lasting change due to insufficient support, lack of follow-through, and competing clinical demands. The approach used in STUN, including practice facilitation and building off of social learning theory, provides tailored support that incorporates a range of techniques to enable integration of evidence-based practices into routine workflows, allowing practices to overcome initial barriers to start-up and sustainment.^51^ Practice facilitation is a complex, multicomponent implementation strategy, and it is unclear whether a singular component of our STUN practice facilitation approach improved outcomes, or whether a dynamic interplay between its many components led to improvement.

By addressing a significant gap in the application of practice facilitation (ie, application to evidence-based practices for unhealthy alcohol use), this study broadens the scope of conditions for which practice facilitation may be beneficial. We demonstrated that practice facilitation can improve the adoption of evidence-based screening and directly improve the adoption of evidence-based counseling, which is associated with improved patient outcomes. Future research should continue to investigate how to optimize the use of practice facilitation for behavioral health conditions, including and beyond unhealthy alcohol use, and in various settings, such as rural or underserved communities, which face unique implementation barriers. The STUN II trial plans to do just that by comparing several implementation strategies and expanding the scope to cover evidence-based screening and interventions for substance use disorder more broadly.^52,53^ Additionally, research on practice facilitation should prioritize identifying the mechanisms of change^54^ generating its effectiveness by (1) exploring the moderators of the association between practice facilitation and adoption (eg, whether practice capacity for quality improvement, implementation climate, or contextual factors moderate the effect to explain the differences between high and low performing practices)^55,56^; (2) investigating the role of context in implementation through codesign efforts^57,58,59^; and (3) evaluating which specific components of practice facilitation—a complex, multi-component implementation strategy—are necessary, sufficient, and most beneficial. Related to exploring potential moderators, separate analyses of STUN data are under way to this end. For example, 10 practices (47.6%) in STUN had at least 1 behavioral health clinician working in the practice, and whether this factor or other key contextual factors, practice capacity for quality improvement, or organizational readiness to change moderate the association between practice facilitation and adoption (in appropriately adjusted analyses) will be useful information.

The proportion of patients with positive screening results in our study (an estimated 13.9%) was lower than national estimates of those reporting risky drinking (often reported approximately 20% or higher).^60^ We suspect that this is partly due to regional variation, as estimates report North Carolina as having lower rates of risky drinking than many other states (approximately 17%-18% for North Carolina).^61^ Further, sources that report on county-level data indicate even lower rates in rural areas (which the STUN practices were skewed toward), with the overall estimate for rural locations in North Carolina approximately 16% and many specific rural North Carolina counties having estimates of 13% to 14% (as observed in our study).^61,62,63^

### Strengths and Limitations

Among its strengths, our study demonstrated that practice facilitation can foster the adoption of evidence-based screening and counseling under adverse conditions. We successfully recruited geographically and demographically diverse practices of varying sizes from across North Carolina. Although there was significant heterogeneity across practices, perhaps somewhat due to variation in resources and baseline capacity, most participating practices showed notable improvements in screening and counseling rates, suggesting that practice facilitation is a durable strategy. The approach is bolstered by the distinctive statewide AHEC network.^43^ While this could limit applicability to states that lack a similar infrastructure, many states have analogous organizations that are equipped to offer similar services (eg, practice-based research networks, statewide collaboratives, clinically integrated networks), and our study demonstrates the successes that can come from developing and utilizing this type of model.

The study also has limitations, many of which were driven by the COVID-19 pandemic. The STUN study launched just before the pandemic, which introduced substantial challenges with recruiting and retaining primary care practices. Eleven of 32 practices withdrew prior to the start of facilitation, which may have resulted in a group of practices that were more highly motivated. We conducted a complementary qualitative study with practice coaches, revealing that several pandemic-related obstacles—such as staffing shortages, financial pressures, and new workflows—required practices to prioritize pandemic management, leaving limited resources for new initiatives such as STUN.^32^ Additionally, nonpandemic barriers emerged, including competing initiatives within practices (such as North Carolina transitioning Medicaid to a new managed care system) and specific challenges related to alcohol screening. Practice facilitators faced constraints as well, often having to conduct meetings virtually rather than on site and in person, which may have reduced engagement and support. However, this could also be seen as a strength, given that remote delivery may be more feasible to support and sustain. The pandemic also prompted a deviation from the original study protocol, which had planned randomization to additional telehealth support (or not) for lower performing practices after 6 months; this component was discontinued as rapid, widespread adoption of telehealth during the COVID-19 pandemic made such randomization infeasible. Hence, the study was not a randomized trial, limiting the ability to infer causality. Additionally, some screening and counseling may occur informally and may not be consistently documented, leading to potential underreporting of adoption outcomes. We also did not collect information about the duration or content of counseling. Next, as noted above, practice facilitation is a complex multicomponent strategy, and the study design does not allow us to determine the association between specific components of practice facilitation and improved implementation outcomes. Delivery of practice facilitation (mode, duration, and frequency) was manually tracked by practice facilitators and may have been subject to self-report bias. Finally, the results are from a single state and may not be representative of other locations. Further, even within North Carolina, certain populations are overrepresented relative to the demographic composition of the state, specifically Black persons and those living in rural areas (because of the focus small-to-medium practices, the practices eligible were skewed toward rural areas).

## Conclusions

In this quality improvement study, practice facilitation was associated with increased adoption of evidence-based screening and counseling for unhealthy alcohol use when provided to small and medium-sized primary care practices. The increase in screening and counseling is projected to substantially reduce the harms of unhealthy alcohol use.

## Notes

Supplement 1. **eFigure 1.** Number and Percentage of Patients Who Screened Positive for Unhealthy Alcohol Use **eFigure 2.** Number and Percentage of Patients Screened, Stratified by Lower vs Higher Baseline Screening Rates

Supplement 2.
Data Sharing Statement
