# Supplementary material for: Screening and Counseling for Unhealthy Alcohol Use in Primary Care Practices
Source: JAMA Netw Open. 2026 Jan 22;9(1):e2553518. doi: 10.1001/jamanetworkopen.2025.53518 (PMC12828630; doi:10.1001/jamanetworkopen.2025.53518)
Supplement: Supplement 1. — eFigure 1. Number and Percentage of Patients Who Screened Positive for Unhealthy Alcohol Use eFigure 2. Number and Percentage of Patients Screened, Stratified by Lower vs Higher Baseline Screening Rates [file jamanetwopen-e2553518-s001.pdf]

Supplementary Online Content

Jonas DE, Riley SR, Brouwer L, et al. Screening and counseling for unhealthy alcohol use in primary care practices. *JAMA Netw Open.* 2026;9(1):e2553518. doi:10.1001/jamanetworkopen.2025.53518

eFigure 1. Number and Percentage of Patients Who Screened Positive for Unhealthy Alcohol Use

eFigure 2. Number and Percentage of Patients Screened, Stratified by Lower vs Higher Baseline Screening Rates

This supplementary material has been provided by the authors to give readers additional information about their work.

© 2026 Jonas DE et al. *JAMA Network Open*.

eFigure 1. Number and Percentage of Patients Who Screened Positive for Unhealthy Alcohol Use

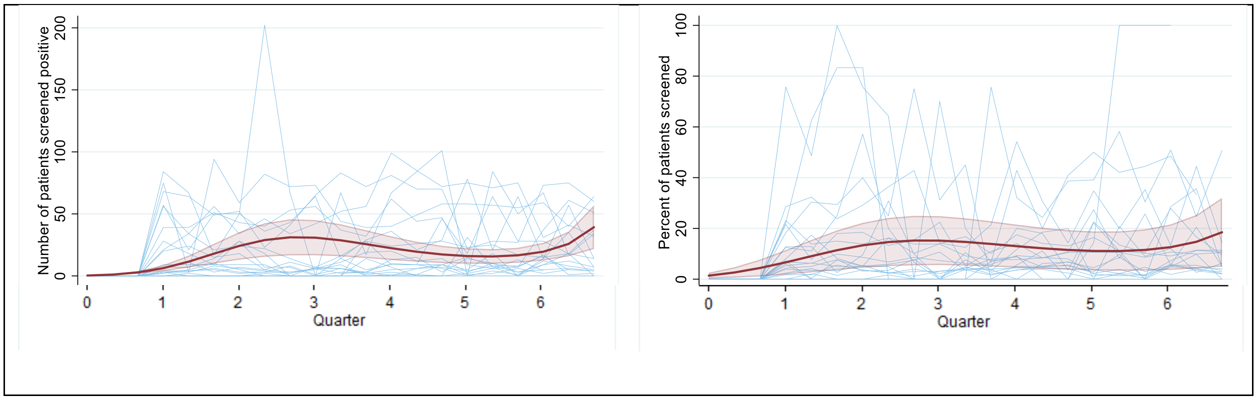

Thin blue lines represent patient counts or percentages for each clinic; wide red line represents the estimated model with pink area surrounding the line representing the 95% confidence bands.
The percentage represents how many had a positive screen out of all adults screened.

© 2026 Jonas DE et al. *JAMA Network Open*.

eFigure 2. Number and Percentage of Patients Screened, Stratified by Lower vs Higher Baseline Screening Rates

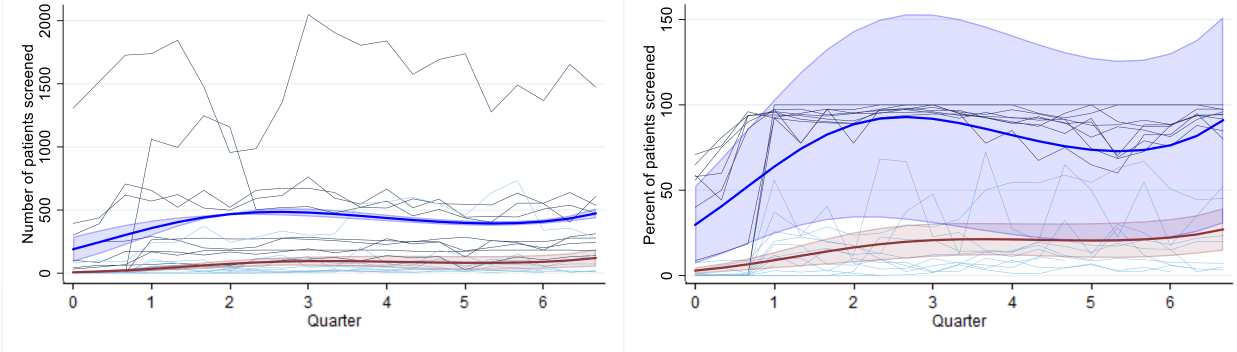

Thin light blue lines represent patient counts or percentages for each clinic with a screening rate of less than 50% in the 3 months prior to the implementation phase; thin dark blue lines represent patient counts or percentages for each clinic with a screening rate of at least 50% in the 3 months prior to the implementation phase; wide red line represents the estimated model for clinics with baseline screening rates of less than 50% with pink area surrounding the line representing the 95% confidence bands; wide blue line represents the estimated model for clinics with baseline screening rates of at least 50% with blue area surrounding the line representing the 95% confidence bands.

© 2026 Jonas DE et al. *JAMA Network Open*.
