# Supplementary material for: Screening and Counseling for Unhealthy Alcohol Use in Primary Care Practices
Source: JAMA Netw Open. 2026 Jan 22;9(1):e2553518. doi: 10.1001/jamanetworkopen.2025.53518 (PMC12828630; doi:10.1001/jamanetworkopen.2025.53518)
Supplement: Supplement 2. — Data Sharing Statement [file jamanetwopen-e2553518-s002.pdf]

Data Sharing Statement

Jonas. Screening and Counseling for Unhealthy Alcohol Use in Primary Care Practices. *JAMA Netw Open*. Published January 22, 2026. doi:10.1001/jamanetworkopen.2025.53518

Data
Data available: Yes
Data types: Deidentified participant data, Data dictionary
How to access data: Data will be made available after publication of main results for each of the aims of the study
When available: beginning date: 07-01-2027

Supporting Documents
Document types: None

Additional Information
Who can access the data: researchers requesting the data when their proposed use has been approved
Types of analyses: secondary analyses of the dataset
Mechanisms of data availability: with investigator support and after approval of a proposal
